# Pip5k1c Loss in Chondrocytes Causes Spontaneous Osteoarthritic Lesions in Aged Mice

**DOI:** 10.14336/AD.2022.0828

**Published:** 2023-04-01

**Authors:** Minghao Qu, Mingjue Chen, Weiyuan Gong, Shaochuan Huo, Qinnan Yan, Qing Yao, Yumei Lai, Di Chen, Xiaohao Wu, Guozhi Xiao

**Affiliations:** ^1^Department of Biochemistry, School of Medicine, Guangdong Provincial Key Laboratory of Cell Microenvironment and Disease Research, Shenzhen Key Laboratory of Cell Microenvironment, Southern University of Science and Technology, Shenzhen, China.; ^2^Shenzhen Hospital (Futian) of Guangzhou University of Chinese Medicine, Shenzhen, Guangdong, China.; ^3^Department of Orthopedic Surgery, Rush University Medical Center, Chicago, IL 60612, USA.; ^4^Research Center for Human Tissues and Organs Degeneration, Institutes of Advanced Technology, Chinese Academy of Sciences, Shenzhen, China.

**Keywords:** Pip5k1c, osteoarthritis, articular chondrocytes, aging

## Abstract

Osteoarthritis (OA) is the most common degenerative joint disease affecting the older populations globally. Phosphatidylinositol-4-phosphate 5-kinase type-1 gamma (Pip5k1c), a lipid kinase catalyzing the synthesis of phospholipid phosphatidylinositol 4,5-bisphosphate (PIP2), is involved in various cellular processes, such as focal adhesion (FA) formation, cell migration, and cellular signal transduction. However, whether Pip5k1c plays a role in the pathogenesis of OA remains unclear. Here we show that inducible deletion of Pip5k1c in aggrecan-expressing chondrocytes (cKO) causes multiple spontaneous OA-like lesions, including cartilage degradation, surface fissures, subchondral sclerosis, meniscus deformation, synovial hyperplasia, and osteophyte formation in aged (15-month-old) mice, but not in adult (7-month-old) mice. Pip5k1c loss promotes extracellular matrix (ECM) degradation, chondrocyte hypertrophy and apoptosis, and inhibits chondrocyte proliferation in the articular cartilage of aged mice. Pip5k1c loss dramatically downregulates the expressions of several key FA proteins, including activated integrin β1, talin, and vinculin, and thus impairs the chondrocyte adhesion and spreading on ECM. Collectively, these findings suggest that Pip5k1c expression in chondrocytes plays a critical role in maintaining articular cartilage homeostasis and protecting against age-related OA.

Osteoarthritis (OA) is a common degenerative joint disease characterized by progressive degeneration of articular cartilage, subchondral sclerosis, synovial inflammation, and osteophyte formation [1]. The clinical symptoms of OA include chronic pain, joint swelling and stiffness, and limited range of motion, leading to disability, psychological distress, and reduced quality of life [2]. The major risk factors for developing OA include aging, joint trauma, obesity, and genetic susceptibility [3]. During the last decade, the global prevalence of OA has rapidly increased, especially in aged populations [4, 5]. For instance, in China, the number of patients with symptomatic OA has increased from 26.1 million to 61.2 million, from 1990 to 2017 [6]. To date, there are no FDA-approved medications that can effectively prevent or delay OA progression due to a limited understanding of OA pathogenesis. Thus, it is highly desirable to investigate the pathological mechanisms underlying OA initiation, development, and progression.

Type 1 phosphatidylinositol 4-phosphate 5-kinases (Pip5k1s) are a group of lipid kinases that phosphorylate the fifth hydroxyl of phosphatidylinositol 4 phosphate (Pi4p) to synthesize phospholipid phosphatidylinositol 4,5-bisphosphate (Pip2) [7]. The latter can serve as a second messenger directly or as a precursor to form other second messengers, such as inositol 1,4,5-triphosphate and diacylglycerol [8]. In mammals, there are three isoforms of Pip5k1 protein, termed Pip5k1a, Pip5k1b, and Pip5k1c [9]. Cumulative evidence has highlighted the pivotal functions of Pip5k1c in a series of physiological processes, such as focal adhesion (FA) formation and dynamics, cell migration, vesicle trafficking, intracellular calcium release, energy metabolism, and cellular signal transduction [10-17]. Moreover, alterations in Pip5k1c expression and/or activation have been linked to several disease conditions, such as osteoporosis, neural dysfunction, obesity, pain hypersensitivity, inflammation, and tumor metastasis [13, 18-21]. In humans, homozygous mutations in the *PIP5K1C* gene cause a rare condition termed lethal congenital contracture syndrome 3 (LCCS3), which is characterized by severe joint contracture, reduced or absent limb movement, and lethality at or soon after birth [22]. In mice, homozygous deficiency of the *Pip5k1c* gene causes early lethality, with a 50% reduction of Pip2 in the brain and impaired synaptic transmission in cortical neurons [23]. Xu and colleagues have reported that the polarization of Pip5k1c induced by integrins is required for the recruitment of neutrophils during inflammatory responses [20]. Zhu et al. have reported that excessive Pip5k1c expression impairs osteoclast formation and bone resorption via enhancing the Pip2 generation [24]. Our previous study has demonstrated an essential role of Pip5k1c, through its expression in mesenchymal stem cells (MSCs), in the control of bone remodeling [25]. Loss of Pip5k1c in MSCs leads to a low turn-over osteopenia-like phenotype in adult mice, by impairing Runx2-mediated osteoblast differentiation and subsequent bone formation [25]. While these studies have clearly indicated the involvement of Pip5k1c in physio-pathological conditions of the musculoskeletal system, whether Pip5k1c plays a role in the pathogenesis of OA remains unknown.

In this study, we demonstrate that inducible deletion of Pip5k1c expression in aggrecan-expressing chondrocytes causes spontaneous OA-like phenotypes in aged (15-month-old) mice, but not in adult (7-month-old) mice. Pip5k1c loss decreases chondrocyte proliferation and increases cell apoptosis in the knee joint articular cartilage of aged mice. Pip5k1c loss inhibits the expression of anabolic extracellular matrix (ECM) proteins and promotes chondrocyte hypertrophic differentiation in aged articular cartilages. Pip5k1c deletion impairs chondrocyte-ECM adhesion partially through downregulation of the expression of several FA proteins.

## MATERIALS AND METHODS

### Animal studies

The generation of *Pip5k1c^fl/fl^* mice was previously described [25]. The *Pip5k1c^fl/fl^* mice were bred with the *Aggrecan^CreERT2^* knock-in transgenic mice to obtain the *Pip5k1c^fl/fl^; Aggrecan^CreERT2^* mice. For inducible deletion of *Pip5k1c* gene in aggrecan-expressing chondrocytes, 2-month-old male *Pip5k1c^fl/fl^; Aggrecan^CreERT2^* mice were intraperitoneally injected with tamoxifen (Sigma T5648, 100 mg/kg per body weight/day, 5 continuous injections). Age-matched male *Pip5k1c^fl/fl^; Aggrecan^CreERT2^* mice were treated with corn oil and served as the control group. All research protocols in this study were approved by the Institutional Animal Care and Use Committees (IACUC) of the Southern University of Science and Technology.

### Micro-computerized tomography

In vivo micro-computerized tomography (µCT) analyses of the knee joint were performed according to our previously established protocol [26, 27]. After sacrifice, the knee joints were collected, fixed in 4% paraformal-dehyde for 24 hours, and scanned using a Skyscan scanner 1276 high-resolution µCT scanner (Bruker, Aartselaar, Belgium) with 60 kVp source and 100 µAmp current with a resolution of 10 µm. Three-dimensional structural reconstructions were performed using the scanned µCT images from each group at the same thresholds. Quantitative µCT parameters, including bone mineral density (BMD) and the volume of calcified meniscus and synovial tissue, were analyzed as previously described [26, 28].

### Histology

The decalcification, dehydration, and paraffin embedding of knee joint samples were performed as previously described [26, 29]. The paraffin-embedded knee joint samples were cut into 5-µm thick sections and stained with Safranin O & Fast Green (SO&FG) (Solarbio, Cat#G1371) as previously described [26, 30]. The severity of OA-like lesions was evaluated using the Osteoarthritis Research Society International (OARSI) scoring system in a double-blinded manner. The Safranin O-positive areas of articular cartilage and growth plate were analyzed by Image J (version 1.53k) as previously described [26]. Representative images were selected based on the mean values of histological scores.

### Quantitative immunofluorescent analyses

For immunofluorescent (IF) staining, 5-µm knee joint sections were hydrated and permeabilized with Immunostaining Permeabilization Solution with Saponin (Beyotime, Cat# P0095) for 5 mins at room temperature (RT), blocked with Immunol Staining Blocking Buffer (Beyotime, Cat# P0102) for 1h at RT, and then incubated with primary antibodies overnight at 4°C. Antibodies used for IF staining in this study were Pip5k1c (Santa Cruz, sc-377061, 1:50), Aggrecan (Abcam, ab36861, 1:200), Col2a1 (Abcam, ab34712, 1:200), Mmp13 (Abcam, ab39012, 1:200), Adamts5 (Abcam, ab41037, 1:200), Col10a1 (Abcam, ab58632, 1:200), Runx2 (Abcam, ab23981, 1:200), Ki67 (CST, 12202S, 1:200), 9EG7 (BD Pharmingen, 553715, 1:200), talin (Abcam, ab110080, 1:200), and vinculin (Santa Cruz, sc-73614, 1:200). After washing in PBS with 0.1% Tween 20, the sections were incubated with Goat anti-Rabbit IgG (H+L) Cross-Adsorbed Secondary Antibody, Alexa Fluor 488 (Invitrogen, Cat# A-11008) (1:400) for 1h at RT. Isotype antibody (Normal Rabbit IgG, Sigma, NI01) controls and secondary antibody-only controls were employed to validate antibody specificity and distinguish genuine target staining from the background. The fluorescent signals in articular cartilages were determined using a Leica SP8 Confocal Microsystems. Representative images were selected based on the mean values of fluorescent signals.

### TUNEL staining

Cell apoptosis was evaluated using the One Step TUNEL Apoptosis Assay Kit (Red Fluorescence) (Beyotime, C1090) as previously described [31, 32].

### In vitro siRNA knockdown experiments

Mouse ATDC5 cells were cultured in DMEM/F12 supplemented with 5% FBS, 1% penicillin and streptomycin, and 1% insulin-transferrin-selenium (Gibco™, Cat# 51500056) to induce chondrogenic differentiation. For in vitro knockdown of Pip5k1c expression, ATDC5 cells were transfected with Pip5k1c siRNA using a Lipofectamine RNAiMAX transfection reagent (Invitrogen, Cat# 13778075) as previously described [25, 33, 34]. ATDC5 cells transfected with negative control siRNA were used as the control group. Cell-ECM adhesion assay was performed according to our previously established protocol [26]. At 48h after siRNA transfection, protein extracts were collected and analyzed by western blotting. Antibodies used for western blotting in this study were Pip5k1c (Santa Cruz, sc-377061, 1:1000), Aggrecan (Abcam, ab36861, 1:1000), Col2a1 (Abcam, ab34712, 1:1000), Tubulin (CWBIO, CW0098, 1:1000), PI3K (CST, 4292S, 1:1000), phosphorylated-PI3K (CST, 4228S, 1:1000), Akt (CST, 4691S, 1:1000), phosphorylated-Akt (CST, 4056S, 1:1000), Erk (CST, 9102S, 1:1000), phosphorylated-Erk (CST, 9101S, 1:1000), integrin β1 (CST, 34971, 1:1000), integrin β3 (CST, 13166S, 1:1000), talin (Abcam, ab110080, 1:1000), and vinculin (Santa Cruz, sc-73614, 1:1000). The Pip5k1c siRNA sequence: 5’ primer-GCGU GCAGUCUGGUGGCAATT, 3’ primer-UUGCCACCA GACUGCACGCTT.

### Statistical Analysis

All mice used in this study were randomly assigned to each group. Statistical analyses were completed using the Prism GraphPad. Results were expressed as mean ± standard deviation (s.d.). Normality of data was tested for all variables using the Kolmogorov-Smirnov (K-S) test. For normally distributed data, a two-tailed unpaired Student’s *t* test was used to assess the differences between the two groups. For non-normally distributed data, an unpaired nonparametric Mann-Whitney test was used to determine the statistical difference between the two groups. A two-way ANOVA test was used for the repeated measurement data from longitudinal in vivo µCT analyses and i*n vitro* chondrocyte-ECM adhesion assay. Differences with *P* < 0.05 were considered statistically significant.

### Data availability

All data generated for this study are available from the corresponding authors upon reasonable request.

## RESULTS

### Generation of inducible chondrocyte-specific Pip5k1c knockout mice

**Figure 1.**
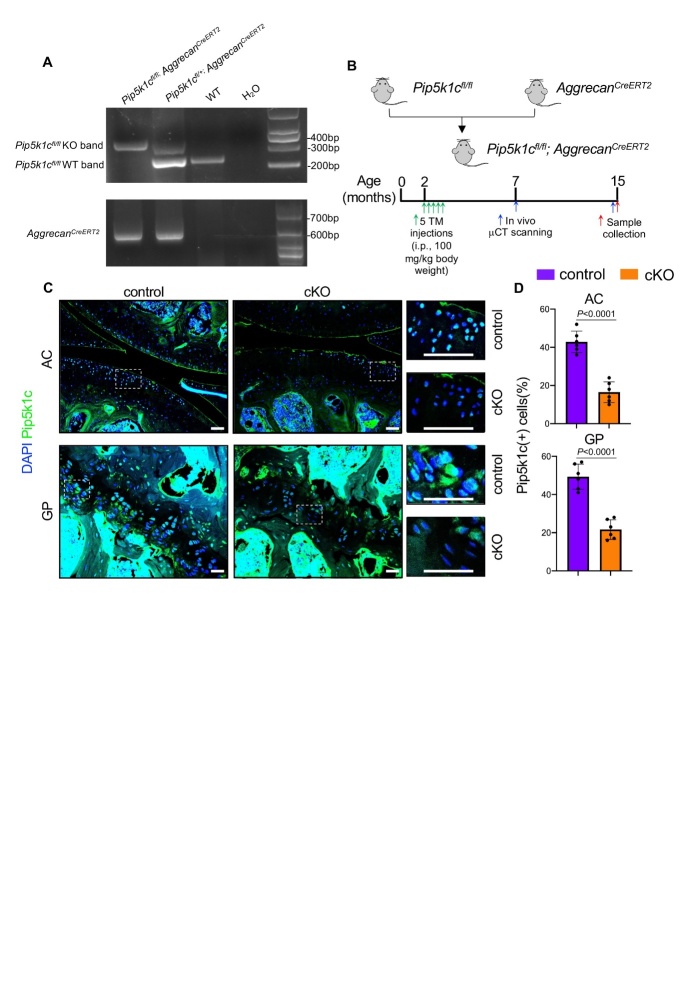
Genetic deletion of Pip5k1c in aggrecan-expressing chondrocytes in adult mice. (A) PCR genotyping using tail DNA. Pip5k1c flox KO band, ~380bp; Pip5k1c flox wildtype (WT) band, ~250bp; Aggrecan^CreERT2^, ~650bp. (B) A schematic diagram illustrating the breeding strategy and experimental design. (C) Immunofluorescent (IF) staining of knee joint sections showing the reduced expression of Pip5k1c in articular cartilage (AC) and growth plate (GP) after TAM injections. White dashed boxes indicate the higher magnification images in the right panels. Scale Bar: 50 mm. (D) Percentages of Pip5k1c-positive cells in AC and GP. N = 6 mice per group. Results are expressed as mean ± standard deviation (s.d.). The exact *P* values are shown in the figures.

To investigate the role of Pip5k1c in chondrocytes, the floxed Pip5k1c (*Pip5k1c^fl/fl^*) mice were bred with *Aggrecan^CreERT2^* transgenic mice to generate *Pip5k1c^fl/fl^*; *Aggrecan^CreERT2^* mice (Fig. 1A, B). For inducible deletion of *Pip5k1c* in aggrecan-expressing chondrocytes, 2-month-old male *Pip5k1c^fl/fl^*; *Aggrecan^CreERT2^* mice were intraperitoneally injected with tamoxifen (TAM, 100 mg kg^-1^ body weight) (hereafter referred to as cKO). Note: Age- and sex-matched *Pip5k1c^fl/fl^*; *Aggrecan^CreERT2^* mice treated with corn oil were used as a control group. At 5 and 13 months after TAM injections, *in vivo* µCT scans were performed to detect structural changes in the knee joints. At 15 months of age, all mice were sacrificed, and the knee joints were collected for further analyses. The deletion of Pip5k1c in chondrocytes was confirmed by immunofluorescent (IF) staining (Fig. 1C). Quantitative analyses showed that the percentages of Pip5k1c-positive cells were decreased by 26.7% and 27.7% in articular cartilage and growth plate, respectively, in cKO mice as compared to those in control mice (*P* < 0.0001, two-tailed unpaired Student’ *t* test) (Fig. 1D).

### Pip5k1c loss causes subchondral bone sclerosis and osteophyte formation in aged mice

**Figure 2.**
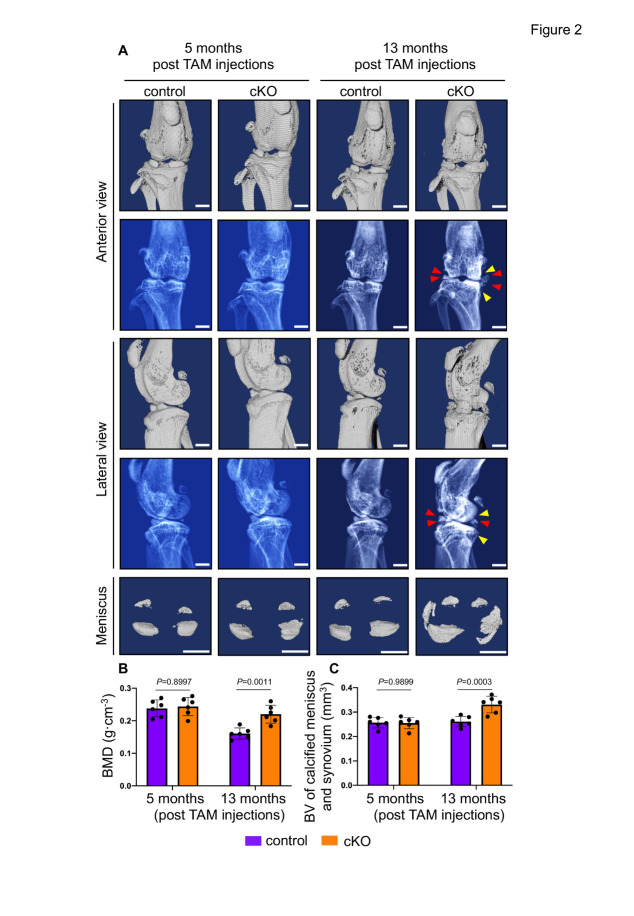
Pip5k1c loss induces subchondral bone sclerosis and osteophyte formation in aged mice. (A) In vivo μCT scans of knee joints from control and cKO mice at 5- and 13-months post-TAM injections. Scale bar, 1 mm. Red arrowheads indicate the formation of osteophytes. Yellow arrowheads indicate subchondral bone sclerosis. (B, C) Quantitative analyses of bone mineral density (BMD) (B) and the volume of calcified meniscus and synovial tissue (C). N = 6 mice per group. Results are expressed as mean ± standard deviation (s.d.). The exact *P* values are shown in the figures.

Results from *in vivo* µCT analyses showed no marked difference in knee joint structure between control and cKO groups at 5 months after TAM injections (Fig. 2A, left panels). Interestingly, we observed a significant increase in subchondral bone sclerosis and osteophyte formation in cKO mice at 13 months after TAM injections (*P* = 0.0011, two-way ANOVA test) (Fig. 2A, right panels). Moreover, quantitative µCT parameters, including bone mineral density (BMD) and bone volume of calcified meniscus and synovium, were comparable between the two groups at 5 months after TAM injections, but were markedly increased in cKO mice at 13 months after TAM injections relative to control group (*P* = 0.0003, two-way ANOVA test) (Fig. 2B, C).

### Pip5k1c loss causes spontaneous OA-like lesions in aged mice

**Figure 3.**
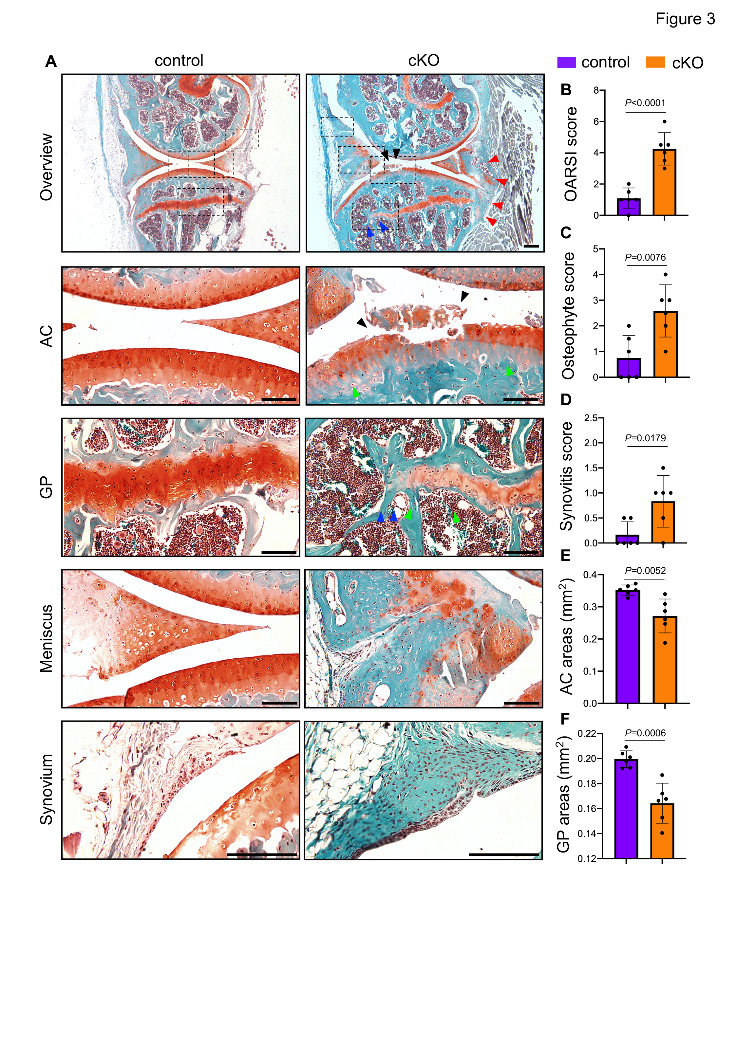
Loss of Pip5k1c in chondrocytes promotes OA-like lesions in aged mice. (A) Representative images of safranin O & fast green (SO&FG)-stained knee joint sections from control and cKO mice at 13 months after TAM injections. Black dashed boxes indicate the higher magnification images of AC, GP, meniscus, and synovium in lower panels. Black arrowheads indicate the degradation of AC. Blue arrowheads indicate the loss of integrity of GP. Red arrowheads indicate the formation of osteophytes. Scale bar, 50 μm. (B) The severity of OA-like lesions was analyzed using the Osteoarthritis Research Society International (OARSI) scoring system. (C, D) Quantitative analyses of safranin O-positive areas in the AC (c) and GP (d). (E, F) Osteophyte score (E) and synovitis score (F) were performed using histological sections. N = 6 mice per group. Results are expressed as mean ± standard deviation (s.d.). The exact *P* values are shown in the figures.

Next, we performed safranin O & fast green (SO&FG) staining on knee joint sections from control and cKO mice at 13 months after TAM injections. Strikingly, the cKO mice displayed a series of severe OA-like lesions, including spontaneous surface fissures of articular cartilage (Fig. 3A, black arrowheads), loss of integrity in growth plate (Fig. 3A, blue arrowheads), loss of safranin O staining in articular cartilage and growth plate (Fig. 3A, green arrowheads), and excessive osteophyte formation (Fig. 3A, red arrowheads). Moreover, meniscus deformation and synovial hyperplasia were observed in cKO mice (Fig. 3A). Quantitative histological analyses revealed significantly higher Osteoarthritis Research Society International (OARSI) scores, osteophyte scores, and synovitis scores in cKO mice as compared with those in control mice (Fig. 3B-D) (*P* < 0.05 for all indicated parameters, two-tailed unpaired Student’s *t* test). In addition, the safranin O-stained cartilage areas were decreased by 22.72% and 20.41% in articular cartilage and growth plate, respectively, in cKO versus control mice (Fig. 3E, F).

**Figure 4.**
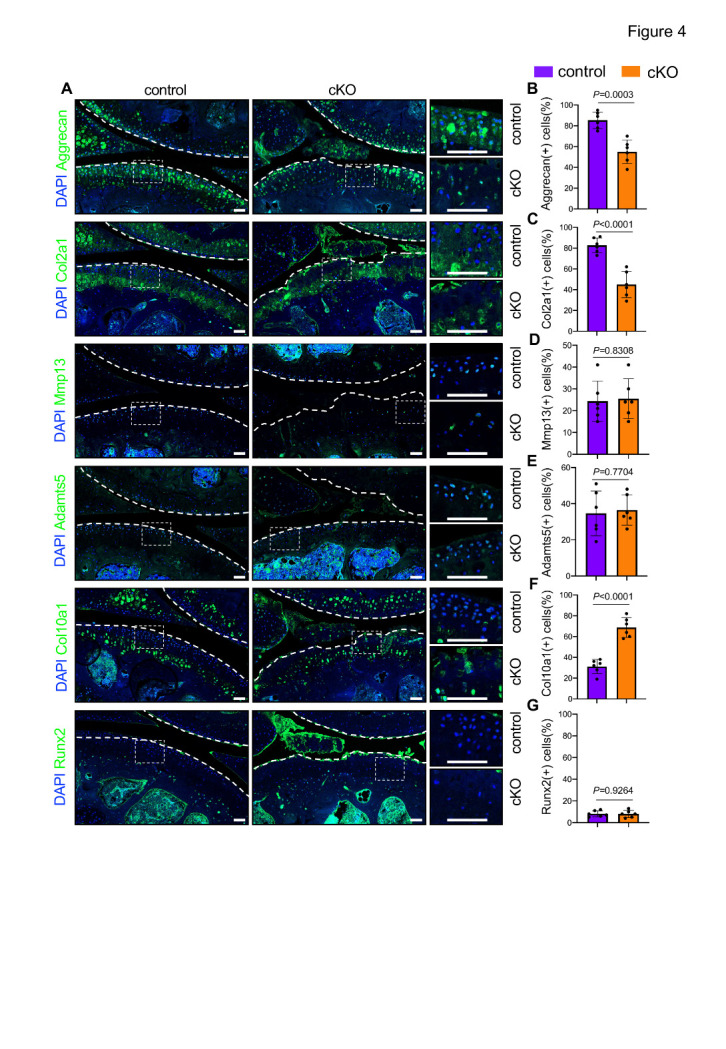
Pip5k1c loss causes ECM degradation and chondrocyte hypertrophic differentiation in aged mice. (A) IF staining for expressions of aggrecan, Col2a1, Mmp13, Adamts5, Col10a1, and Runx2 using knee joint sections from control or cKO mice at 13 months after TAM injections. White dashed boxes indicate the higher magnification images in the right panels. White dashed lines indicate the cartilage surfaces. Scale bar: 50 µm. (B-G) Quantitative analyses of the percentages of aggrecan-, Col2a1-, Mmp13-, Adamts5-, Col10a1-, and Runx2-positive cells in AC. N = 6 mice per group. Results are expressed as mean ± standard deviation (s.d.). The exact *P* values are shown in the figures.

### Pip5k1c loss promotes ECM degradation and chondrocyte hypertrophic differentiation in aged mice

IF staining analyses revealed that Pip5k1c loss significantly decreased the expression levels of anabolic ECM proteins, including aggrecan and Col2a1, in the articular cartilage of cKO mice (Fig. 4A-C). Notably, the percentages of aggrecan- and Col2a1- positive cells were decreased by 30.3%and 37.7%, respectively, in cKO versus control cartilages (*P* < 0.001, two-tailed unpaired Student’ *t* test) (Fig. 4A-C). Interestingly, we found that the expression levels of catabolic ECM enzymes, including Mmp13 and Adamts5, were comparable between the two groups (Fig. 4A, E, F). We next determined the expression levels of chondrocyte hypertrophic markers, including Col10a1 and Runx2, by IF staining analyses. Results showed that, while Col10a1 was barely detectable in the superficial and middle layers of the articular cartilages in control mice, its expression was dramatically upregulated in these areas in cKO mice (*P* < 0.0001, two-tailed unpaired Student’ *t* test) (Fig. 4A, F). Interestingly, Runx2 expression was not markedly increased in the articular cartilages of cKO mice compared with that in control mice (Fig. 4A, G).

### Pip5k1c loss decreases chondrocyte proliferation and increases chondrocyte apoptosis in aged mice

**Figure 5.**
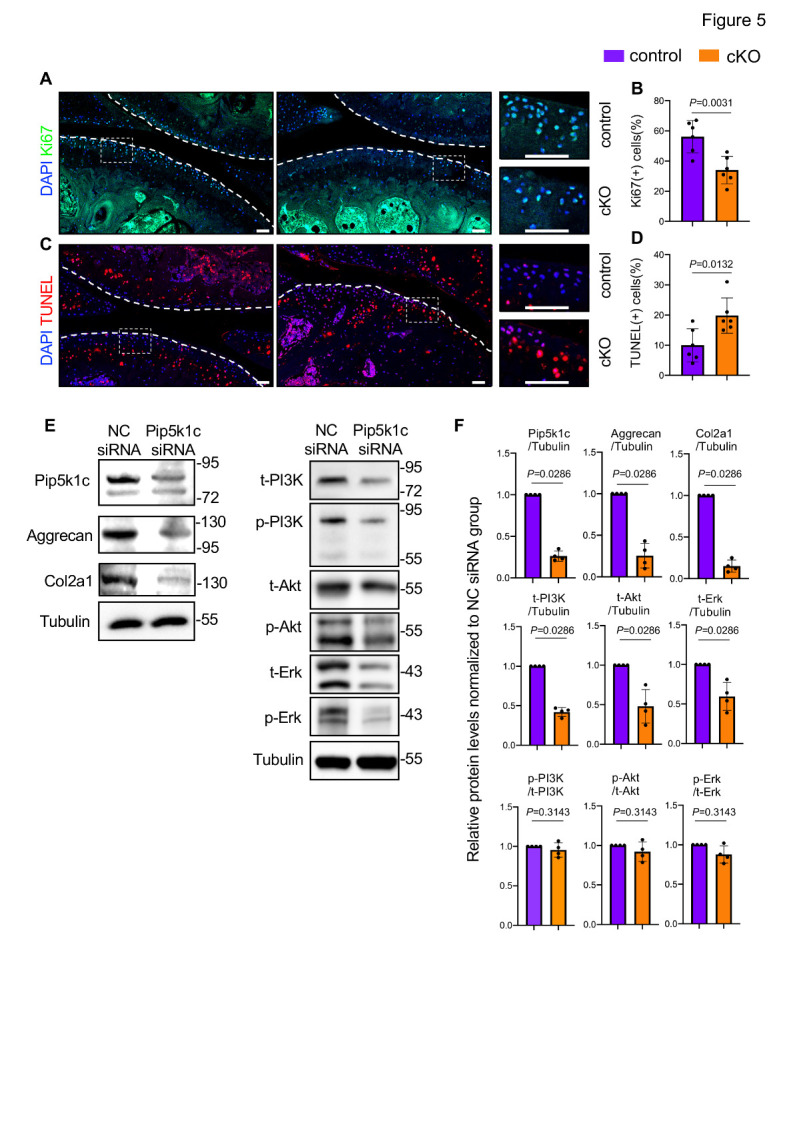
Pip5k1c loss inhibits chondrocyte proliferation and induces chondrocyte apoptosis in aged mice. (A) IF staining for expression of Ki67 using knee joint sections from control or cKO mice at 13 months after TAM injections. White dashed boxes indicate the higher magnification images in the right panels. White dashed lines indicate the cartilage surfaces. Scale bar: 50 µm. (B) Quantitative data of (A). (C) Fluorescent TUNEL staining. Scale bar: 50 µm. (D) Quantitative data of (d). (E) Western blotting. Protein extracts were isolated from cultured ATDC5 cells transfected with negative control (NC) siRNA or Pip5k1c siRNA and subjected to western blot analyses with indicated antibodies. t: total; p: phosphorylated. (F) Relative protein levels normalized to the NC siRNA group. In vitro siRNA knockdown experiments were independently repeated four times. Results are expressed as mean ± standard deviation (s.d.). The exact *P* values are shown in the figures.

We further performed IF staining of cell proliferation marker Ki67 to assess whether the proliferative activity of articular chondrocytes could be affected by Pip5k1c deficiency. In control mice, Ki67 was strongly detected in cells of the superficial and middle layers of articular cartilage (Fig. 5A). However, the percentage of Ki67-positive chondrocytes was decreased by 22.17% in these areas of cKO mice compared to that in control mice (Fig. 5B) (56.17±10.72% in control group vs 34±9.08% in Pip5k1c-cKO group, *P* = 0.0031, two-tailed unpaired Student’ *t* test). Moreover, results from the terminal deoxynucleotidyl transferase-mediated nick-end labelling (TUNEL) staining revealed that Pip5k1c loss markedly increased the number of apoptotic chondrocytes in the superficial and middle layers of articular cartilage in cKO mice (*P* = 0.0132, two-tailed unpaired Student’ *t* test) (Fig. 5C, D). In vitro studies from cultured ATDC5 cells showed that siRNA knockdown of Pip5k1c dramatically reduced the protein level of aggrecan and Col2a1 in these cells (*P* < 0.05, unpaired nonparametric Mann-Whitney test) (Fig. 5E, F). Moreover, Pip5k1c siRNA treatment significantly downregulated the total and phosphorylated protein levels of PI3K, Akt, and Erk (*P* < 0.05 for all indicated parameters, unpaired nonparametric Mann-Whitney test), without affecting the phosphorylated/total ratios of these proteins (Fig. 5E, F).

**Figure 6.**
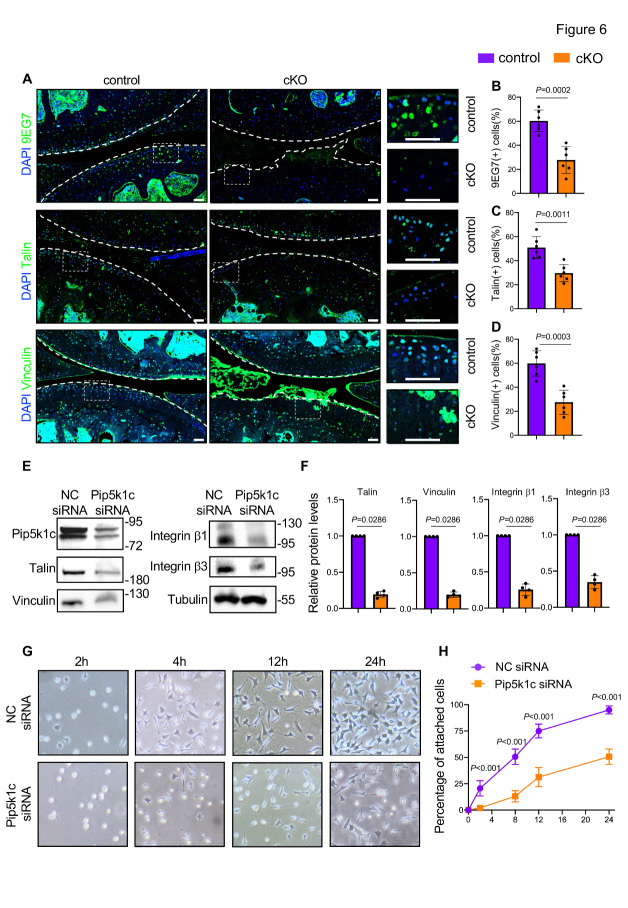
Pip5k1c loss reduces expression of FA proteins and impairs chondrocyte-ECM adhesion. (A) IF staining for expressions of activated integrin β1 (9EG7), talin, and vinculin using knee joint sections from control or cKO mice at 13 months after TAM injections. White dashed boxes indicate the higher magnification images in the right panels. White dashed lines indicate the cartilage surfaces. Scale bar: 50 µm. (B-D) Quantitative data of (a). (E) Western blotting. Protein extracts were isolated from cultured ATDC5 cells which had been transfected with NC siRNA or Pip5k1c siRNA. (F) Relative protein levels of talin, vinculin, integrin β1, and integrin β3 in ATDC5 cells transfected with NC siRNA or Pip5k1c siRNA. (g) Representative images of attachment and spreading of ATDC5 cells on type II collagen-coated surfaces after transfection of NC siRNA or Pip5k1c siRNA. (H) Percentages of attached cells. In vitro experiments were independently repeated at least three times. Results are expressed as mean ± standard deviation (s.d.). The exact *P* values are shown in the figures.

### Pip5k1c loss reduces the expression of FA proteins and impairs chondrocyte-ECM adhesion

Previous studies have reported a pivotal role of Pip5k1c in controlling the FA formation [10]. Thus, we determined the expressions of FA-related molecules, including activated integrin β1 (9EG7), talin, and vinculin, in articular cartilage by quantitative IF analyses (Fig. 6A). Results showed that the percentages of 9EG7-, talin- and vinculin-positive cells were all dramatically decreased in articular cartilages of cKO mice when compared with those in control mice (Fig. 6A-D) (*P* < 0.001 for all indicated parameters, two-tailed unpaired Student’ *t* test). Consistently, siRNA knockdown of Pip5k1c in ATDC5 cells resulted in decreased protein expressions of talin, vincular, integrin β1, and integrin β3 (*P* < 0.05 for all indicated parameters, unpaired nonparametric Mann-Whitney test) (Fig. 6E, F). Furthermore, siRNA knockdown of Pip5k1c drastically impaired the attachment and spreading of ATDC5 cells on collagen-II-coated surfaces in vitro (*P* < 0.001 for Pip5kc1 siRNA group vs NC siRNA group, two-way ANOVA test) (Fig. 6G, H).

## DISCUSSION

Although the complex molecular mechanisms underlying the onset and progression of OA remain incompletely understood, cumulating evidence has pointed to the fact that aging itself is the most prominent risk factor contributing to OA development [35, 36]. Results from clinical studies have shown that the incidence and severity of OA are much higher in aged populations when compared with younger populations [37, 38]. OA is the leading cause of disability in the population aged over 65 and is associated with comorbid disorders, higher mortality, and reduced quality of life [39]. In this study, we provide convincing evidence that genetic deletion of Pip5k1c in chondrocytes causes multiple spontaneous OA lesions, including articular cartilage damage, subchondral sclerosis, synovial inflammation, and osteophyte formation in aged mice. We find that Pip5k1c loss inhibits chondrocyte proliferation, and induces chondrocyte hypertrophy, apoptosis, and ECM degradation. Pip5k1c loss reduces the expression of several key FA proteins and impairs the chondrocyte-ECM adhesions. Notably, this is the first demonstration of the crucial role of Pip5k1c in the maintenance of cartilage homeostasis to protect against aging-induced OA.

In healthy articular cartilages, the ECM forms a complex scaffold comprising collagens, proteoglycans, water content, and fibrous proteins, which not only endows the articular cartilage with unique biomechanical properties but also provides chondrocytes a distinctive microenvironment for maintaining their cellular homeostasis [40]. During OA development, articular chondrocytes undergo abnormal hypertrophic differentiation, leading to reduced synthesis of anabolic ECM proteins, excessive production of chondrocyte hypertrophic marker Col10a1, and upregulations of ECM-degrading enzymes, such as Mmp13 and Adamts4/5 [1]. Runx2 is a well-known transcriptional factor for its role in promoting chondrocyte hypertrophy and OA development [41-45]. Our recent study has demonstrated that Pip5k1c regulates the expression level of Runx2 protein, but not its mRNA, though mediating calcium/calmodulin-dependent protein kinase 2 (CaMK2) and cytoplasmic Ca^2+^ levels, in MSCs [25]. Interestingly, in this study, we find that Pip5k1c deletion enhances chondrocyte hypertrophic differentiation and ECM degradation without upregulation of Runx2 expression in articular chondrocytes. Molecular mechanisms whereby Pip5k1c loss induces chondrocyte hypertrophy and ECM degradation in articular cartilage require further investigations.

Unlike other types of arthritis, OA usually develops slowly over many years [46]. Under physiological conditions, the turnover of aggrecan takes up to 25 years, whereas the half-life of type II collagen ranges from several decades to up to 400 years [47]. Interestingly, results from this study suggest that genetic ablation of Pip5k1c in aggrecan-expressing chondrocytes causes OA-like lesions in aged (15-month-old) mice, but not in adult (7-month-old) mice, which highly mimic the pathological features of OA in humans. We find that Pip5k1c loss dramatically decreases the expression levels of anabolic ECM proteins, including Col2a1 and aggrecan, in the articular cartilages, without upregulating the expression of catabolic enzymes Mmp13 and Adamts5. This finding suggests that Pip5k1c loss impairs ECM homeostasis via suppressing the anabolic activities of articular chondrocytes rather than promoting ECM catabolism, which might partially explain the slowly progressive OA-like phenotypes in the cKO mice. Moreover, whether the function of Pip5k1c is compensated by other Pip5k1s, such as Pip5k1a and Pip5k1b, to delay OA progression in adult cKO mice needs to be determined in future studies.

By utilizing genetically modified animal models, several key molecules and signaling pathways responsible for cartilage degradation and OA onset and progression have been identified, which involve Wnt/β-catenin, Runx2, FGF, miRNAs, Ampk, mTOR, and FA signaling pathways [26, 28, 29, 48-55]. For instance, Zhu and coworkers have reported that sustained activation of β-catenin in articular chondrocytes leads to multiple OA-like phenotypes, including cartilage loss, subchondral remodeling, and chondrophyte/osteophyte formation probably by upregulation of Runx2 [48]. Genetic deletion of Runx2 in chondrocytes decelerates the progression of surgically induced OA, whereas overexpression of Runx2 exerts the opposite effects [49, 50]. In addition, our previous study has demonstrated that mechanical loading activates the mTOR signaling pathway and promotes OA development in mouse temporomandibular joints [54]. In this study, we demonstrate that Pip5k1c loss induces dysregulation of several key signaling pathways involved in cartilage homeostasis and survival by downregulating the protein expressions of PI3K, Akt, and Erk in chondrocytes. Moreover, Pip5k1c loss significantly inhibits the proliferative activity of articular chondrocytes while inducing chondrocyte apoptosis and ECM degradation in articular cartilage. Collectively, these findings suggest that Pip5k1c expression may play a critical role in maintaining cartilage homeostasis via regulating cellular signaling transductions.

Results from this study suggest that Pip5k1c loss induces cartilage degradation and OA-like lesions through, at least in part, impairing the chondrocyte-ECM adhesion. We provide several lines of evidence to support this notion. First, Pip5k1c loss significantly decreases the expression levels of key FA-related proteins, including talin and vinculin, and inhibits the activation of integrin β1 in articular chondrocytes in aged mice. Second, siRNA knockdown of Pip5k1c downregulates the protein expressions of talin, vinculin, integrin β1, and integrin β3 in ATDC5 chondrogenic cells. Third, we demonstrate that Pip5k1c loss drastically impairs the adhesion ability of chondrocytes on collagen type II in vitro. These findings, along with results from our previous study that FA-related molecule plays an essential role in preserving the integrity of articular cartilage to protect against OA damages [26], indicate a potential mechanism that involves Pip5k1c and its interactions with the FA signaling pathway in the pathogenesis of OA onset and progression. It is well established that Pip5k1c catalyzes the phosphorylation of Pi4p to synthesize Pip2, the latter can be further phosphorylated by PI3K to form the second messenger phosphatidylinositol 3,4,5-trisphosphate (Pip3) and activate Akt signaling [56]. Moreover, Pip2 interacts with several key FA proteins, such as FA kinase (FAK), talin, and vinculin, to regulate the FA assembly and dynamics [57-60]. Akt can not only phosphorylate Pip5k1c specifically at serine 555 to regulate Pip5k1c-talin interaction and focal adhesion dynamics [61], but can also act downstream of Pip3 to mediate numerous cellular processes [62]. Whether Pip5k1c loss reduces the expression of FA proteins and impairs chondrocyte-ECM adhesion through downregulating PI3K/Akt activity needs to be determined in future studies.

It should be noted that Pip5k1c loss causes synovitis-like changes, including hyperplasia of synovial lining cells and inflammatory infiltration, in the knee joints of aged mice. Our previous study has demonstrated that *Aggrecan^CreERT2^* is highly active in articular chondrocytes, but not in cells of the synovium [26]. Thus, the observed alterations in the synovium are indirect results of Pip5k1c deletion in chondrocytes by *Aggrecan^CreERT2^*. It is well known that OA is a whole joint disease. Loss of Pip5k1c in chondrocytes impairs ECM homeostasis, proliferation, and adhesion, and promotes cell apoptosis in articular cartilages, which may subsequently induce synovial inflammation, for instance, by changing the micro-environment of the joint. The underlying mechanisms need further investigation in future studies.

We acknowledge that this study has several limitations. First, we did not determine the expression level of Pip5k1c in human cartilages. Whether Pip5k1c expression is altered in human OA cartilages needs to be determined. Second, since articular cartilage is a weight-bearing tissue, it will be interesting to determine if and how Pip5k1c loss in chondrocytes affects the OA development in instability-induced OA models, such as the destabilization of the medial meniscus model. Third, while our results clearly show that Pip5k1c loss induces multiple spontaneous osteoarthritic lesions in aged mice, whether overexpression of Pip5k1c in mouse chondrocytes can exert protective effects against aging-induced OA onset and progression remains to be investigated. In conclusion, our study demonstrates a vital role of Pip5k1c expression in aggrecan-expressing chondrocytes in the regulation of the articular cartilage homeostasis in mice.
